# Immunoglobulin A Nephropathy in a SARS-CoV-2-Positive Patient With Coexistent Metabolic Syndrome

**DOI:** 10.7759/cureus.28719

**Published:** 2022-09-03

**Authors:** Pranjal Kalita, Biswajit Dey, Jaya Mishra, Iadarilang Tiewsoh, Vandana Raphael

**Affiliations:** 1 Pathology, North Eastern Indira Gandhi Regional Institute of Health and Medical Sciences (NEIGRIHMS), Shillong, IND; 2 Internal Medicine, North Eastern Indira Gandhi Regional Institute of Health and Medical Sciences (NEIGRIHMS), Shillong, IND

**Keywords:** covid-19, serum creatinine, sars-cov-2, metabolic syndrome, iga nephropathy

## Abstract

SARS-CoV-2 viral infection though primarily affects the respiratory system, but concurrent renal involvement is been reported in the medical literature. Acute kidney injury (AKI) is a common finding in SARS-CoV-2-positive patients. An isolated case of IgA nephropathy in a SARS-CoV-2 virus-infected patient has been already reported in the medical literature. Incidence of metabolic syndromes is on the rise considering the change in lifestyle and food habits and the global pandemic of obesity. Renal manifestations of metabolic syndrome are myriad with IgA nephropathy being an occasional manifestation in such patients. We reported a case of IgA nephropathy in a patient in her fourth decade of life diagnosed as metabolic syndrome with concomitant SARS-CoV-2 infection that progressed to chronic kidney damage (CKD) subsequently. In this case report, we postulate that cytokine storm along with hypoxemia secondary to SARS-CoV-2 infection may accelerate the declining renal function however further studies are necessary to confirm this hypothesis considering the rarity of such cases.

## Introduction

Immunoglobulin A (IgA) nephropathy first described by Berger and Hinglais in 1968 was defined as the presence of dominant or co-dominant mesangial IgA immune deposits [1]. Metabolic disease is on the rise considering the changes in lifestyle and dietary habits and the global pandemic of obesity [2]. Concurrent IgA nephropathy in a patient with type 2 diabetes mellitus is the commonest medical non-diabetic kidney disease (NDKD) in Asian patients [3] Along with type 2 diabetes mellitus hypertension in IgA nephropathy patients may lead to slow and progressive end-stage renal disease (ESRD) [4]. Recent severe acute respiratory syndrome coronavirus 2 (SARS-CoV-2) infection is a pandemic that primarily affects the lungs however its renal manifestations are slowly getting recognized [4]. Renal biopsies show a wide range of findings ranging from endothelial injuries, collapsing variant of focal segmental glomerulosclerosis (FSGS), and acute tubular necrosis (ATN) [5]. SARS-CoV-2-positive cases diagnosed with acute kidney injury (AKI) were initially rare; however recent studies indicate incidence of AKI in >50% of patients admitted to intensive care unit (ICU) and in >20% of the hospitalized patients positive for SARS-CoV-2 infection [6]. Chronic kidney disease (CKD) is an infrequent renal manifestation in SARS-CoV-2-positive patients with an incidence ranging from 0.7% to 2.9% [7]. We report a rare case of concurrent IgA nephropathy in a SARS-CoV-2-positive patient in her fourth decade of life progressing rapidly to CKD who was a diagnosed as a case of metabolic syndrome at presentation and also hypothesize a probable explanation of her rapid decline in renal function.

## Case presentation

A 38-year-old female presented to the outpatient department (OPD) with complaints of generalized weakness and abdominal discomfort for one week. There was no history of decreased urine output, blood in urine, burning micturition, swelling of the lower limbs, or any bodily rashes. The patient was afebrile and did not complain of cough or chest pain. On examination, the patient was conscious and oriented. On examination, her blood pressure was 144/100 mm Hg, mean arterial pressure was 114.6 mm Hg, pulse rate was 84 beats per minute, respiratory rate was 20 breaths per minute, and SpO_2_ was 94%. On elaborating on the history of medication intake, the patient revealed that she was advised oral hypoglycaemic, statins, and antihypertensive; however, the patient also states that she was not taking the medications regularly. She was not vaccinated for the SARS-CoV-2 virus.

The patient tested positive for the SARS-CoV-2 virus by reverse transcription-polymerase chain reaction (RT-PCR) and was admitted in accordance with the institute protocol. The patient was treated conservatively with tablet doxycycline 100 mg twice daily, vitamin C, and zinc capsules. The patient’s laboratory samples were evaluated (Table 1).

**Table 1:** Laboratory parameters of the patient at presentation Hb: hemoglobin; TLC: total leucocyte count; DLC: differential leucocyte count; N: neutrophil; L: lymphocyte; M: monocyte; E: eosinophil; HbA1c: glycated hemoglobin; HDL: high-density lipoprotein; LDL: low-density lipoprotein; IL-6: interleukin-6; ESR: erythrocyte sedimentation rate

Laboratory tests	Results
Hemoglobin (Hb)	10.8 g/dL
Total leucocyte count (TLC)	12.6 × 10^9^/L
Differential leucocyte count (DLC)	N87 L07 M04 E02
Platelets	282 × 10^9^/L
Fasting blood sugar	130 mg/dL
Postprandial blood sugar	210 mg/dL
HbA_1c_	6.5%
Urea	63 mg/dL
Serum creatinine	1.8 mg/dL
Estimated glomerular filtration rate (eGFR)	33 mL/min/1.73m^2^
Triglyceride	707 mg/dL
Cholesterol	264 mg/dL
High-density lipoprotein (HDL)	42.4 mg/dL
Low-density lipoprotein (LDL)	179.1 mg/dL
Interleukin-6 (IL-6)	198.3 pg/mL
D-dimer	615 pg/mL
Erythrocyte sedimentation rate (ESR)	40 ng/mL
Urine analysis	
Protein	3+
Blood	3+
24-h urine protein	3604.01 mg/24-h

Ultrasound studies showed chronic medical renal parenchymal disease. The patient underwent a renal biopsy considering the nephrotic range proteinuria along with concomitant deranged biochemical and urine routine examination findings. The patient biopsy report was awaited and was conservatively started on subcutaneous injection of regular insulin 15-20 minutes before a meal, anti-hypertensive drugs along with statins. The patient took discharge against medical advice and did not return for follow-up.

**Figure 1:**
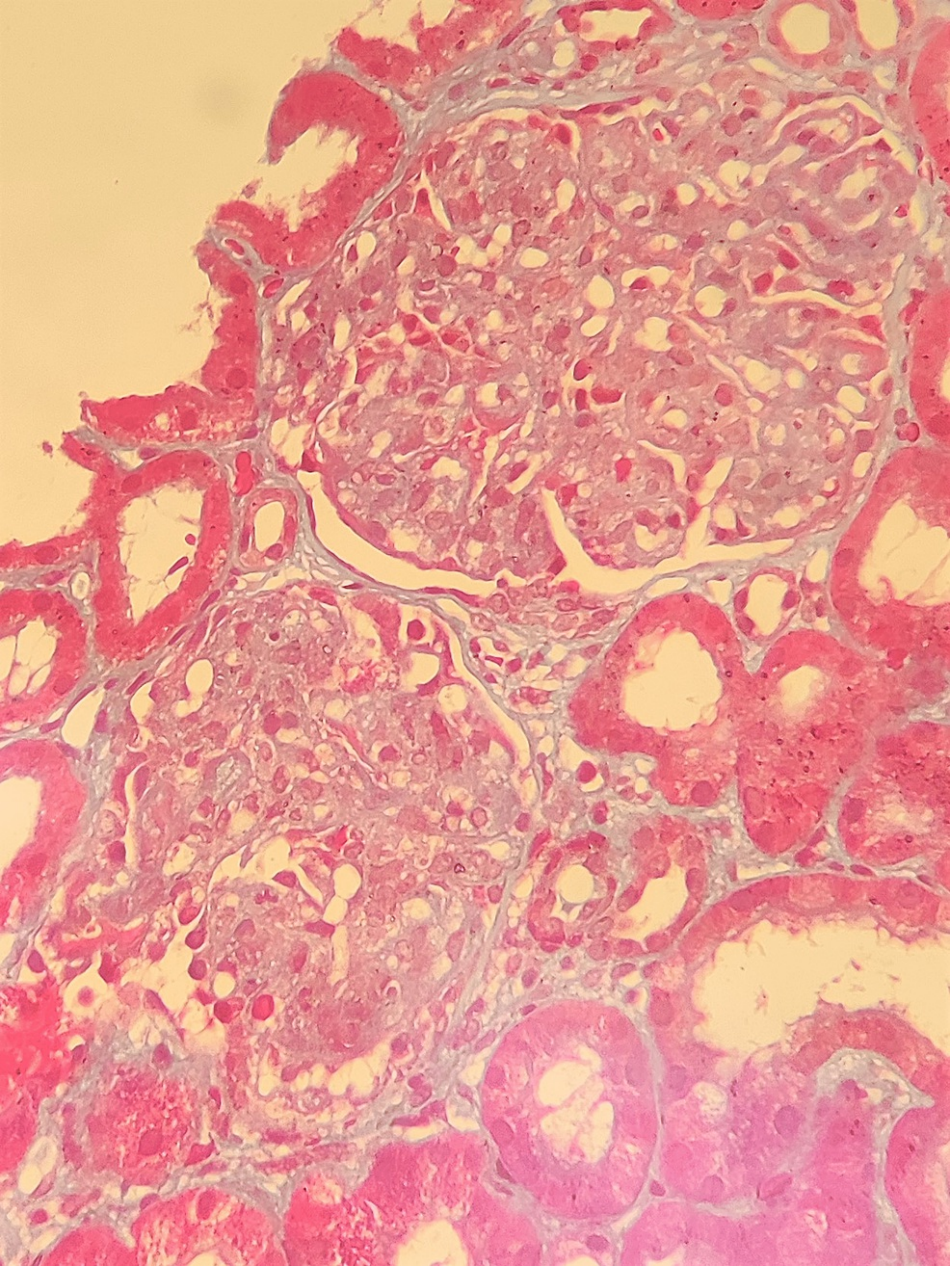
Glomerulus shows mesangial matrix expansion, hypercellularity, endocapillary hypercellularity, focal obliteration of endocapillary loops, thickening of glomerular basement membrane, and lobular accentuation (PAS, 200×) PAS: periodic acid-Schiff

 Light microscopy of hematoxylin and eosin (H&E), periodic acid-Schiff (PAS), Masson's trichrome (MT), and Jones methenamine silver (JMS) stained slides examined by two nephropathologists independently showed a total of 10 intact glomeruli. The glomerular compartment showed glomerular basement membrane (GBM) thickening along with mesangial matrix expansion and hypercellularity, obliteration of the endocapillary lumen, and lobular accentuation (Figure 1). Two glomeruli were completely sclerosed. No crescents were noted. Tubules show evidence of necrosis (25-50%), thyroidisation, and intraluminal debris. The interstitium shows fibrosis and lymphoplasmacytic infiltrate. Blood vessels showed arteriolar hyalinosis and mild medial hypertrophy. The mesangial hypercellularity, endocapillary hypercellularity, segmental sclerosis, tubular atrophy/interstitial fibrosis, and crescents (MEST-C) score for the patient was M1E1S1T0C0.

**Figure 2:**
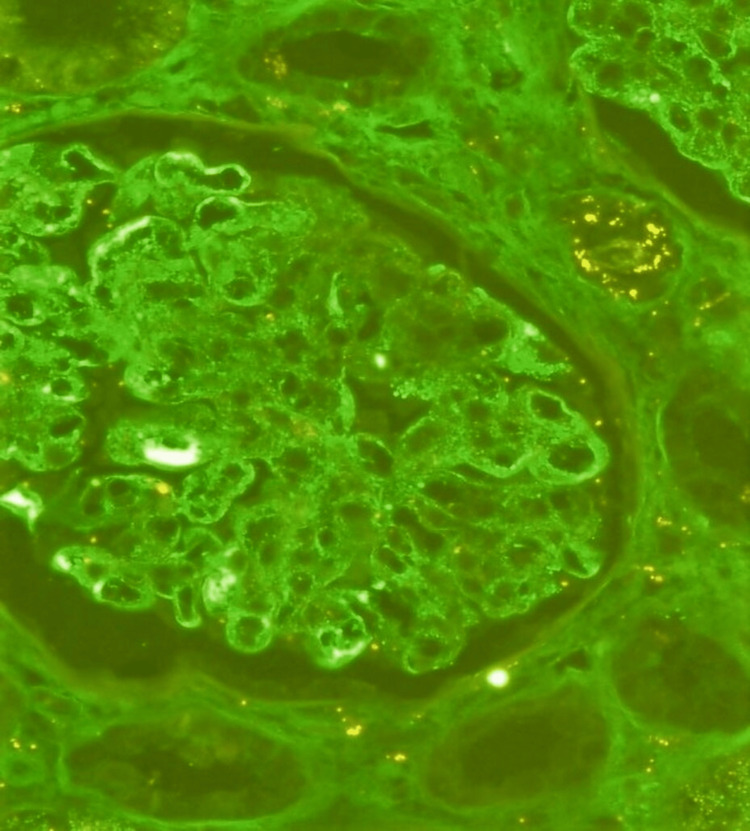
Immunofluorescence studies show IgA (3+) immunostain deposit in mesangium and glomerular basement membrane (200×).

Immunofluorescence studies for IgA, IgG, IgM, C3, kappa, and lambda showed 3+ granular mesangial immunostain deposits for IgA, kappa, and lambda (Figure 2). Immunostain IgG, IgM, and C3 revealed no immune deposits.

Transmission electron microscopy (TEM) studies on the renal biopsy tissue received in glutaraldehyde failed to reveal any viral inclusion particle. Mesangial electron-dense deposits were noted.

In view of the light microscopy, immunofluorescence, and electron microscopy findings a diagnosis of IgA nephropathy with concomitant diabetic and hypertensive changes was noted.

The patient was lost to follow-up and presented after 3.5 months from the initial presentation. She complained of increased weakness. On examination, her various organ systems were unremarkable. The patient's vitals were monitored and BP was 166/117 mm Hg and mean arterial pressure (MAP) was 182.3 mm of Hg. Biochemical examination revealed proteinuria and haematuria; however, the fasting blood sugar (FBS) and postprandial blood sugar (PPBS) values of 83 mg/dL and 101 mg/dL along with HbA1c levels of 5.9% revealed control of the diabetic state. The kidney function test showed urea levels of 72 mg/dL and serum creatinine levels of 2.2 mg/dL. The estimated glomerular filtration rate (eGFR) calculated by modification of diet in renal disease (MDRD) was 27 mL/min/1.73m^2^. The lipid profile of the patient had improved but was still slightly deranged with a triglyceride level of 245 mg/dL, a cholesterol level of 205 mg/dL, HDL of 44 mg/dL, and LDL of 160 mg/dL. An ophthalmology examination of the fundus did not reveal any diabetic retinopathy. She tested negative for hepatitis B, hepatitis C, and human immunodeficiency virus. The patient's histopathology reports were also reviewed.

The patient was continued on subcutaneous injection of regular insulin, an antihypertensive agent (losartan 50 mg), statin (rosuvastatin 0 mg), and omega-3 fatty acid capsules, were prescribed. No immunosuppressants or steroid-containing medications were used prior to or after the biopsy.

The patient was followed up after one month; her glycaemic control, as well as blood pressure level and lipid profile, was within the permissible limit. However, kidney function tests still revealed raised serum creatinine (1.9 mg/dL). The patient was asymptomatic at the last visit and was continued on the same medications and advised follow-up.

## Discussion

Non-diabetic kidney disease was a common finding in patients with type 2 diabetes mellitus and may lead progressively to ESRD [8]. According to a study by Mak et al., among type 2 diabetic patients with NDKD, the incidence of IgA nephropathy was 59% [9]. Gans et al. postulated an interesting common pathogenic pathway in the development of diabetic glomerulosclerosis and IgA nephropathy. Alterations in type 2 diabetic patients’ glomeruli facilitate the localization of immunological complexes of galactose-deficient IgA1 implicated in the pathogenesis of IgA nephropathy [10,11]. Concomitant hypertension in IgA nephropathy patients is a common finding with incidence ranging from 31% to 63.3% [4]. 

National Cholesterol Education Programme-Adult Treatment Panel III (NCEP ATP III) defined “metabolic syndrome” as a syndrome that satisfied three or more of the five criteria [12]. The definition of metabolic syndrome was revised by Zimmet et al. in 2005, which included ethnic group-specific cut-off based on central obesity measured on the basis of waist circumference as an important parameter in the diagnosis of “metabolic syndrome” [13]. In the same study, the risk of type 2 diabetes mellitus was found to be higher in Asian Indian patients with waist circumference of greater than 85 cm in men and values of greater than 80 cm in women [13].

The presence of concomitant hyperglycemia, hypertension along with hypertriglyceridemia in patients with metabolic syndrome presenting with concomitant IgA nephropathy had been explained by various authors [14].

Virus-associated glomerulopathy was commonly associated with hepatitis B, hepatitis C, and human immune deficiency viral infections [5]. SARS CoV-2 virus utilizes human angiotensin-converting enzyme 2 (ACE2) and transmembrane protease serine 2 (TMPRSS2) receptors localized primarily to the proximal tubules, collecting duct, and to a lesser extent to the mesangial cells, podocytes and lining parietal epithelium of the kidneys [5]. CD 147 localized to the proximal tubules had also been implicated in the pathogenesis of SARS-CoV-2 viral infection [15]. Renal manifestations of SARS-CoV-2 virus infection are variable and involved various compartments of the kidney with manifestations ranging from minimal change disease (MCD), collapsing focal segmental glomerulosclerosis (FSGS), crescentic glomerulonephritis, thrombotic microangiopathies and IgA nephropathy [5,7]. Hematuria and proteinuria following SARS-CoV-2 virus infection leading to acute kidney injury (AKI) had been reported in various case reports [16]. Metabolic syndrome and SARS-CoV-2 virus infection have been independently reported to cause IgA nephropathy in patients which may ultimately lead to ESRD [17].

In our present study, the waist circumference of greater than 35 inches for women was absent whereas elevated blood pressure, fasting triglyceride and blood sugar, and decreased fasting high-density lipoprotein level in the patient satisfied four of the five requisite criteria required for the diagnosis of metabolic syndrome in accordance with NCEP ATP III parameters.

The tubular compartment showed features of acute tubular necrosis (ATN), a finding commonly noted in viral nephropathies but not entirely specific. TEM studies in our case failed to reveal any viral-like particle but were consistent with renal findings for a diagnosis of IgA nephropathy.

There has been a recent surge in the studies relating to the pathogenesis and novel biomarkers in IgA nephropathy including dendrin. Increased dendrin expression is associated with milder histopathological lesions and better kidney functions [18]. IgA nephropathy (IgAN) and IgA vasculitis, are part of a similar clinical spectrum, occurring with COVID-19 [19]. However, the patient in the present study had no signs or symptoms of any vasculitic lesions. The authors of the present study were of the opinion that IgA nephropathy in the patient was a result of type 2 diabetes mellitus aided with hypertension and deranged lipid profile priming the kidney for immune complex deposition. Rapid progression to CKD and histological findings of tubular necrosis were a result of the indirect effects of hypoxemia and cytokine storm secondary to SARS-CoV-2 viral infection as described in an article by Ahmadian et al. and also partly attributed to the patient's history of non-adherence to the treatment regime [20]. Although steroids could have been used in the treatment modality lack of studies to confirm its beneficial role was a reason for no steroids being used in our patient.

## Conclusions

Metabolic syndrome characterized by a constellation of obesity, deranged lipid profile, raised blood sugar levels, and hypertension may lead to IgA nephropathy. The cytokine storm, hypoxemia secondary to SARS-CoV-2 infection, may accelerate the declining renal function in patients with concomitant SARS-CoV-2 infection with metabolic syndrome and IgA nephropathy however more studies are required to confirm this hypothesis.
